# A Global Overview of COVID-19 Research in the Pediatric Field: Bibliometric Review

**DOI:** 10.2196/24791

**Published:** 2021-07-23

**Authors:** Alice Monzani, Francesco Tagliaferri, Simonetta Bellone, Giulia Genoni, Ivana Rabbone

**Affiliations:** 1 Division of Pediatrics Department of Health Sciences Università del Piemonte Orientale Novara Italy; 2 Pediatric and Neonatal Intensive Care Unit Maggiore della Carità University Hospital Novara Italy

**Keywords:** COVID-19, SARS-CoV-2, children, pediatrics, bibliometric review, publications, research, literature, review

## Abstract

**Background:**

Since the beginning of the COVID-19 pandemic, a great number of papers have been published in the pediatric field.

**Objective:**

We aimed to assess research around the globe on COVID-19 in the pediatric field by bibliometric analysis, identifying publication trends and topic dissemination and showing the relevance of publishing authors, institutions, and countries.

**Methods:**

The Scopus database was comprehensively searched for all indexed documents published between January 1, 2020, and June 11, 2020, dealing with COVID-19 in the pediatric population (0-18 years). A machine learning bibliometric methodology was applied to evaluate the total number of papers and citations, journal and publication types, the top productive institutions and countries and their scientific collaboration, and core keywords.

**Results:**

A total of 2301 papers were retrieved, with an average of 4.8 citations per article. Of this, 1078 (46.9%) were research articles, 436 (18.9%) were reviews, 363 (15.8%) were letters, 186 (8.1%) were editorials, 7 (0.3%) were conference papers, and 231 (10%) were categorized as others. The studies were published in 969 different journals, headed by *The Lancet*. The retrieved papers were published by a total of 12,657 authors from 114 countries. The most productive countries were the United States, China, and Italy. The four main clusters of keywords were pathogenesis and clinical characteristics (keyword occurrences: n=2240), public health issues (n=352), mental health (n=82), and therapeutic aspects (n=70).

**Conclusions:**

In the pediatric field, a large number of articles were published within a limited period on COVID-19, testifying to the rush to spread new findings on the topic in a timely manner. The leading authors, countries, and institutions evidently belonged to the most impacted geographical areas. A focus on the pediatric population was often included in general articles, and pediatric research about COVID-19 mainly focused on the clinical features, public health issues, and psychological impact of the disease.

## Introduction

With its massive and rapid spread, the COVID-19 pandemic has been an unprecedented challenge for health care systems worldwide [1]. At the same rate, the research community has been extremely prolific, with a considerable amount of scientific papers published within a very short time, in an effort to address all aspects of disease management in a timely manner. Many journals are offering fast-track publication for COVID-19–related papers. The availability of reviewers for an expedited review process has been called for. In other words, the editorial process that usually characterizes the research on a disease has been sped up and amplified in this exceptional circumstance.

Infection by SARS-CoV-2 seems to be milder in the pediatric population than in the elderly [2], and the relative protection of children against the severe forms of COVID-19 is a major point of interest in the comprehension of the pathogenetic mechanisms of the disease [3]. Therefore, research on COVID-19 in the pediatric field has experienced a strong increase. The very high number of continuously published papers and the speed at which research on this topic has been generated makes it extremely difficult to stay updated with such a rapidly evolving knowledge base. In this regard, a bibliometric analysis of the current literature on pediatric COVID-19 can help clinicians stay updated with emerging and swiftly evolving scientific outcomes. Bibliometric analysis is the attempt to quantitatively assess the current literature on a certain topic, allowing researchers to acquire knowledge about research trends and providing insights into the contribution of a particular country or institution to that topic, as well as data on coauthorship and collaboration [4].

Therefore, we performed a bibliometric analysis of the papers on COVID-19 in the pediatric field published in the first half of 2020 to assess research on this topic on a global scale, identify publication trends, and provide some hints on the gap of knowledge to be filled by future research.

## Methods

The Scopus database was comprehensively searched for all the indexed documents published between January 1, 2020, and June 11, 2020, dealing with COVID-19 in the pediatric population (0-18 years of age). The keywords used were *COVID* or *coronavirus* and *pediatric* or *child* or *children* or *adolescent*$ in the title or abstract. We used only these terms to conduct a broad search that would ensure the inclusion of relevant literature. The inclusion criteria were papers that (1) were published after the first report of COVID-19 from the Wuhan government on December 31, 2019, and (2) matched the search keywords. As COVID-19 was first reported in China and a fairly large number of research papers were written in Chinese, language was not limited during the retrieval process.

The literature retrieval group consisted of 3 trained professionals. We used the export feature of the search engine to retrieve data for further processing. All extracted literature entries were exported into Microsoft Excel (Microsoft Corp) for screening and selection. The reviewers (AM, FT, GG, SB) independently screened the titles, abstracts, and, if ambiguous, full texts for the inclusion of articles. Discrepancies were resolved through discussions among them and with a fifth reviewer (IR) in case of difficulties in reaching an agreement. The reviewers independently conducted information extraction from the included papers. Discrepancies were similarly resolved through discussion among the reviewers.

For studies that fulfilled the inclusion criteria, the following information was extracted: authors, affiliations, and country of origin (when there was more than one author, the corresponding author’s information was used), journal, publication date, publication type, citation, and abstract. The abstract and title of every record retrieved were screened to determine which studies should be assessed further.

We performed explorative data analysis for:

- Total number of papers to measure global productivity;
- Total citations to assess the relevance of an author, institution, or country;
- Journal and publication types to assess topic dissemination;
- Scientific collaboration between authors, institutions, and countries to show how they related to others;
- Core keywords to show clusters of research topics.

The intracountry and intercountry collaboration indices were presented as single-country publication (SCP) and multiple-country publication (MCP), according to the country of the corresponding author, and the timing of the first COVID-19 case reported for each country was highlighted as well.

A machine learning bibliometric methodology was applied to evaluate the distribution of each factor. The *bibliometrix* R package with its Biblioshiny web interface was used [5].

No ethics approvals were considered necessary as this was a literature-based study.

## Results

### General Data

A total of 2301 papers were retrieved. Out of them, 1078 (46.9%) were research articles, 436 (18.9%) were reviews, 363 (15.8%) were letters, 186 (8.1%) were editorials, 7 (0.3%) were conference papers, and 231 (10%) were categorized as others.

### Citation Analysis

The retrieved articles had 11,063 citations with an average of 4.8 citations per article. Of the retrieved papers, 869 (37.8%) were cited at least once. The 10 most frequently cited articles are shown in Table 1. The top-ranking paper (n=1255 citations) was published in *The New England Journal of Medicine* and was focused on the clinical characteristics of COVID-19 in China.

Table 2 shows the distribution of the prevalence of the retrieved articles according to the number of citations.

**Table 1:** List of the 10 most cited articles about COVID-19 in the pediatric population.

Authors and reference	Title	Journal	Publication date	Total citations, n (%)
Guan et al [6]	Clinical characteristics of coronavirus disease 2019 in China	*The New England Journal of Medicine*	April 30, 2020	1255 (11.3)
Chan et al [7]	A familial cluster of pneumonia associated with the 2019 novel coronavirus indicating person-to-person transmission: A study of a family cluster	*Lancet*	February 15, 2020	735 (6.6)
Mehta et al [8]	COVID-19: Consider cytokine storm syndromes and immunosuppression	*Lancet*	March 13, 2020	324 (2.9)
Wu et al [9]	A new coronavirus associated with human respiratory disease in China	*Nature*	February 3, 2020	309 (2.8)
Wu et al [10]	Risk factors associated with acute respiratory distress syndrome and death in patients with coronavirus disease 2019 pneumonia in Wuhan, China	*JAMA Internal Medicine*	March 13, 2020	291 (2.6)
Chen et al [11]	Clinical characteristics and intrauterine vertical transmission potential of COVID-19 infection in nine pregnant women: A retrospective review of medical records	*Lancet*	February 12, 2020	275 (2.5)
Lai et al [12]	Severe acute respiratory syndrome coronavirus 2 (SARS-CoV-2) and coronavirus disease-2019 (COVID-19): The epidemic and the challenges	*International Journal of Antimicrobial Agents*	February 12, 2020	213 (1.9)
Xu et al [13]	Evolution of the novel coronavirus from the ongoing Wuhan outbreak and modeling of its spike protein for risk of human transmission	*Science China Life Sciences*	January 21, 2020	153 (1.4)
Dong et al [14]	Epidemiology of COVID-19 among children in China	*Pediatrics*	June 1, 2020	134 (1.2)
Lu et al [15]	SARS-CoV-2 infection in children	*The New England Journal of Medicine*	April 23, 2020	128 (1.2)

**Table 2:** Distribution of published papers about COVID-19 in the pediatric population according to the number of citations.

Number of citations	Articles, n (%)
>100	17 (0.74)
50-100	25 (1.09)
20-49	70 (3.04)
5-19	202 (8.78)
<5	1989 (86.44)

### Journal Analysis

The studies were published in 969 different journals. *The Lancet* headed the list with a total number of 29 publications, followed by the *Journal of Medical Virology* (n=26) and *Science of the Total Environment* (n=25). In analyzing the publications’ reference lists, the most cited sources were *The Lancet* (n=2336 citations), followed by *The New England Journal of Medicine* (n=1835), and the *Journal of American Medical Association* (n=1017).

### Author Analysis

A total of 12,657 authors contributed to these papers, with a mean of 5.5 authors per document. Out of this total, 239 were authors of single-authored documents and 12,418 were authors of multiauthored documents.

### Country Analysis

The retrieved papers were published by authors from 114 countries. The most productive country in the COVID-19 research field in pediatrics was the United States, with 178 publications, followed by China, with 138 publications, and Italy, with 87 publications. Table 3 shows the top 10 productive countries.

**Table 3:** The top 10 productive countries publishing on the topic of COVID-19 in the pediatric population.

Country	Articles, n	SCP^a^, n	MCP^b^, n	First reported case^c^
United States	178	134	44	January 30, 2020
China	138	119	19	December 31, 2019
Italy	87	70	17	February 20, 2020
India	30	23	7	March 2, 2020
France	26	21	5	February 7, 2020
United Kingdom	23	10	13	January 28, 2020
Canada	22	10	12	January 27, 2020
Iran	19	15	4	February 19, 2020
Korea	18	14	4	January 20, 2020
Australia	17	11	6	January 25, 2020

^a^SCP: single-country publication.

^b^MCP: multiple-country publication.

^c^Source: WHO Coronavirus (COVID-19) Dashboard [16].

### Institution Analysis

The published articles came from 4919 institutions, with the top 10 institutions accounting for 614 (26.7%) papers. The Huazhong University of Science and Technology was the most productive institution, with 117 documents, followed by Harvard Medical School (n=82 publications) and the University of Oxford (n=67). Table 4 displays the top 10 productive institutions.

**Table 4:** The top 10 productive institutions publishing on the topic of COVID-19 in the pediatric population.

Institution	Country	Articles, n (%)
Huazhong University of Science and Technology	China	117 (5.1)
Harvard Medical School	United States	82 (3.6)
University of Oxford	United Kingdom	67 (2.9)
University of Washington	United States	63 (2.7)
University of California	United States	61 (2.6)
University of Toronto	Canada	56 (2.4)
Tehran University of Medical Sciences	Iran	47 (2.0)
University College London	United Kingdom	45 (2.0)
Universal Scientific Education and Research Network	–^a^	39 (1.7)
University of Melbourne	Australia	37 (1.6)

^a^Not applicable.

### Collaboration Analysis

**Figure 1:**
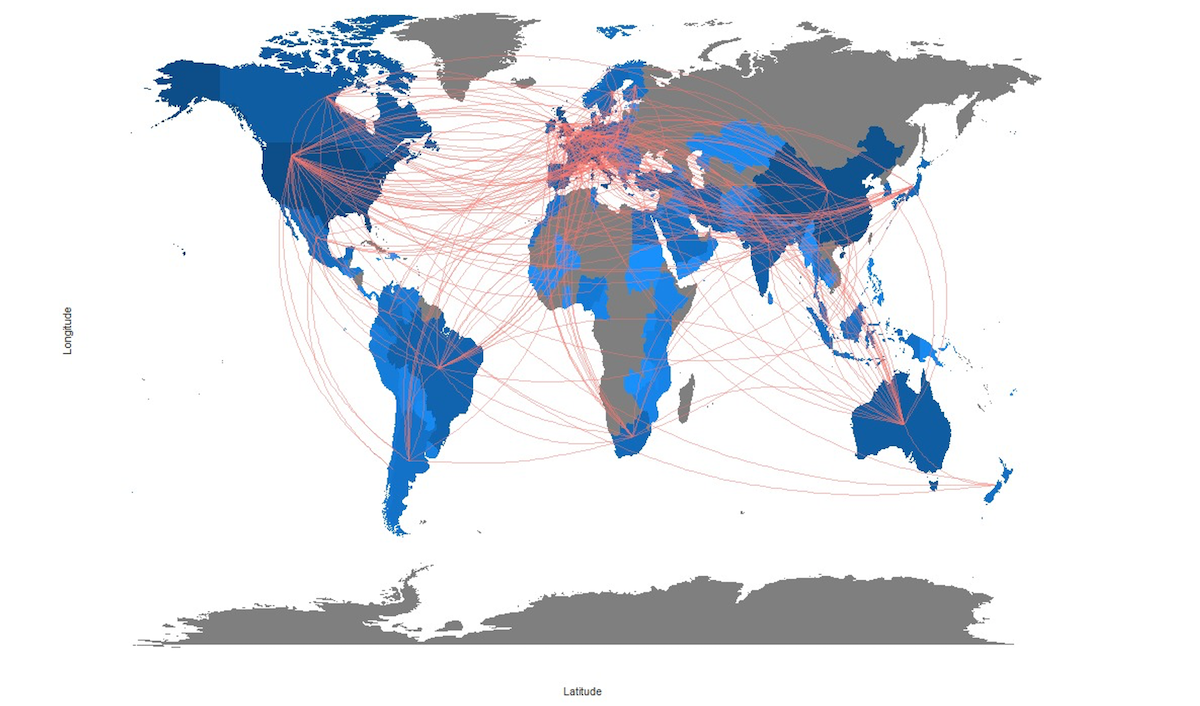
Collaboration map between countries in publications on COVID-19 in the pediatric population.

Figure 1 shows the country collaboration analysis. The network diagram showed that China and the United States were the leaders in COVID-19 research in cooperation with other countries (each node represents a country, node size corresponds to publication number, connecting lines represents country cooperation, and line thickness indicates collaboration frequencies). Overall, developed countries had greater collaboration networks than developing territories.

### Keyword Analysis

Overall, the three most common keywords were *COVID-19* with 880 occurrences, *coronavirus* with 368 occurrences and *Sars-Cov2* with 294 occurrences. Multimedia Appendix 1 shows a word cloud for the most common keywords of the retrieved papers

When keywords were clustered into themes, four main clusters emerged: pathogenesis and clinical characteristics (keyword occurrence: n=2240), public health issues (n=352), mental health (n=82), and therapeutic aspects (n=70).

## Discussion

### Principal Findings

In this bibliometric review, we aimed to provide a comprehensive portrait of published research on COVID-19 in the pediatric population. The bibliometric approach properly fits the aim of representing all scientific publications on a certain topic in a defined time frame. Therefore, this method has been recently used by many authors to depict the state of current knowledge about COVID-19 under different perspectives [17-27], and we used it to focus on the pediatric population.

The most impressive result was the large number of articles published within a limited period on a single topic. Most were research articles, but, notably, more than 15% of the total published papers were letters, testifying to the rush to quickly spread even preliminary results and personal perspectives [28]. The low rate of conference papers was probably due to the fact that most of the national and international conferences scheduled in that period were cancelled in compliance with social distancing measures.

The citation analysis showed that only about one-third of the published papers were cited at least once, suggesting that the short time frame and the rapidly increasing amount of literature likely lowered the possibility of a paper being cited. However, the top-cited paper reached a high number of citations in a very short time.

The leading authors, countries, and institutions evidently belonged to the most impacted geographical areas, as already pointed out in previous general bibliometric analyses.

The keyword analysis, as well as the top-cited articles, revealed that a focus on the pediatric population was often included in general articles about COVID-19 as well. Analyzing the keyword clusters, it was observed that pediatric research about COVID-19 mainly focused on the clinical features of the disease and public health issues. Notably, mental health–related topics were of outstanding interest, even overcoming therapeutic aspects. Psychosocial challenges and opportunities for children with chronic health conditions during lockdown, such as digital approaches to remote pediatric health care delivery, represented a mainstream topic in the publication trends during the pandemic [29,30]. This, along with the lack of emergency preparedness–related topics among the most recurrent keywords, could suggest that COVID-19 research in pediatrics more frequently addressed the psychological impact of lockdown, per the milder clinical course of the disease among children, and unlike what emerged in a similar bibliometric analysis outside the pediatric area [17].

Taken together, our findings demonstrate that scientists around the world, facing the unprecedented emergency of the COVID-19 pandemic, felt compelled to publish their findings and opinions with the aim to contribute to the evolving knowledge base as soon as new evidence emerged. This occurred even in the pediatric field, which has up to now not been so seriously involved in the COVID-19 emergency.

This process was facilitated by high-impact medical journals opening special issues or columns for COVID-19 [31], some publishers waiving publication fees and providing free access to article content, and many journals opting to fast track peer review. It would be desirable that some of these positive aspects implemented in the current difficult times would become common practice. Nonetheless, in such an accelerated publishing process, it should be of great importance to keep the quality of research papers high, which is essential to spread valuable information [22,32]. In the future, it would be interesting to compare this first 6-month publication rush to later publication attitudes about the COVID-19 topic in the pediatric field.

### Limitations

Our study has some limitations that need to be acknowledged, pertaining mainly to the intrinsic bias of having considered only studies indexed in Scopus within a limited time frame, knowing that results may differ had other databases been included or the time period extended. However, this bibliometric analysis of COVID-19 publications in the pediatric field offers a global overview of what has been published on the topic, which allows for the identification of possible gaps in knowledge for new lines of research. In particular, the Scopus database was chosen for its reliability, wide coverage of scientific production, and daily updates. We decided to search only one database mainly to avoid duplicate results. Moreover, as this bibliometric analysis addressed a very recent and rapidly evolving topic, we preferred to search only one reliable database to overcome gaps in the promptness of updates from different sources. Finally, we preferred Scopus as our main data source since it provides data analysis for publications and citations, and allows the results to be sorted according to the number of citations.

Another limitation consists of the limited time frame of our analysis. Using a different methodological approach, such as a living systematic review, would allow for a real-time update on the rising evidence in such a rapidly evolving field. As a result, it would be possible for authors to constantly monitor gaps in knowledge, which they could try to fill with future research.

### Conclusion

In conclusion, a substantial number of papers have been published on the topic of COVID-19 in the pediatric field. It would be advisable to carry on implementing the positive changes to publication policies that emerged during the COVID-19 pandemic, which will aid in providing solid evidence to inform and support clinical and public health decision making.

## Appendix

Multimedia Appendix 1 Word cloud of the most frequently used keywords in papers about COVID-19 in the pediatric population.

## Abbreviations

MCP: multiple-country publication
SCP: single-country publication

## References

[1] WHO Health Emergency Dashboard.

[2] De Luca Carmen Dolores, Esposito E, Cristiani L, Mancino E, Nenna R, Cortis E, Midulla F (2020). Covid-19 in children: A brief overview after three months experience. Paediatr Respir Rev.

[3] Cristiani L, Mancino E, Matera L, Nenna R, Pierangeli A, Scagnolari C, Midulla F (2020). Will children reveal their secret? The coronavirus dilemma. Eur Respir J.

[4] Ellegaard O, Wallin J (2015). The bibliometric analysis of scholarly production: How great is the impact?. Scientometrics.

[5] Aria M, Cuccurullo C (2017). bibliometrix : An R-tool for comprehensive science mapping analysis. Journal of Informetrics.

[6] Guan Wei-Jie, Ni Zheng-Yi, Hu Yu, Liang Wen-Hua, Ou Chun-Quan, He Jian-Xing, Liu Lei, Shan Hong, Lei Chun-Liang, Hui David S C, Du Bin, Li Lan-Juan, Zeng Guang, Yuen Kwok-Yung, Chen Ru-Chong, Tang Chun-Li, Wang Tao, Chen Ping-Yan, Xiang Jie, Li Shi-Yue, Wang Jin-Lin, Liang Zi-Jing, Peng Yi-Xiang, Wei Li, Liu Yong, Hu Ya-Hua, Peng Peng, Wang Jian-Ming, Liu Ji-Yang, Chen Zhong, Li Gang, Zheng Zhi-Jian, Qiu Shao-Qin, Luo Jie, Ye Chang-Jiang, Zhu Shao-Yong, Zhong Nan-Shan, China Medical Treatment Expert Group for Covid-19 (2020). Clinical Characteristics of Coronavirus Disease 2019 in China. N Engl J Med.

[7] Chan Jf, Yuan S, Kok K, To Kk, Chu H, Yang J, Xing F, Liu J, Yip Cc, Poon Rw, Tsoi H, Lo Sk, Chan K, Poon Vk, Chan W, Ip Jd, Cai J, Cheng Vc, Chen H, Hui Ck, Yuen K (2020). A familial cluster of pneumonia associated with the 2019 novel coronavirus indicating person-to-person transmission: a study of a family cluster. The Lancet.

[8] Mehta P, McAuley Df, Brown M, Sanchez E, Tattersall Rs, Manson Jj (2020). COVID-19: consider cytokine storm syndromes and immunosuppression. The Lancet.

[9] Wu C, Zhao S, Yu B, Chen Y, Wang W, Song Z, Hu Y, Tao Z, Tian J, Pei Y, Yuan M, Zhang Y, Dai F, Liu Y, Wang Q, Zheng J, Xu L, Holmes Ec, Zhang Y (2020). A new coronavirus associated with human respiratory disease in China. Nature.

[10] Wu Chaomin, Chen Xiaoyan, Cai Yanping, Xia Jia'an, Zhou Xing, Xu Sha, Huang Hanping, Zhang Li, Zhou Xia, Du Chunling, Zhang Yuye, Song Juan, Wang Sijiao, Chao Yencheng, Yang Zeyong, Xu Jie, Zhou Xin, Chen Dechang, Xiong Weining, Xu Lei, Zhou Feng, Jiang Jinjun, Bai Chunxue, Zheng Junhua, Song Yuanlin (2020). Risk Factors Associated With Acute Respiratory Distress Syndrome and Death in Patients With Coronavirus Disease 2019 Pneumonia in Wuhan, China. JAMA Intern Med.

[11] Chen H, Guo J, Wang C, Luo F, Yu X, Zhang W, Li J, Zhao D, Xu D, Gong Q, Liao J, Yang H, Hou W, Zhang Y (2020). Clinical characteristics and intrauterine vertical transmission potential of COVID-19 infection in nine pregnant women: a retrospective review of medical records. The Lancet.

[12] Lai Chih-Cheng, Shih Tzu-Ping, Ko Wen-Chien, Tang Hung-Jen, Hsueh Po-Ren (2020). Severe acute respiratory syndrome coronavirus 2 (SARS-CoV-2) and coronavirus disease-2019 (COVID-19): The epidemic and the challenges. Int J Antimicrob Agents.

[13] Xu Xintian, Chen Ping, Wang Jingfang, Feng Jiannan, Zhou Hui, Li Xuan, Zhong Wu, Hao Pei (2020). Evolution of the novel coronavirus from the ongoing Wuhan outbreak and modeling of its spike protein for risk of human transmission. Sci China Life Sci.

[14] Dong Yuanyuan, Mo Xi, Hu Yabin, Qi Xin, Jiang Fan, Jiang Zhongyi, Tong Shilu (2020). Epidemiology of COVID-19 Among Children in China. Pediatrics.

[15] Lu Xiaoxia, Zhang Liqiong, Du Hui, Zhang Jingjing, Li Yuan Y, Qu Jingyu, Zhang Wenxin, Wang Youjie, Bao Shuangshuang, Li Ying, Wu Chuansha, Liu Hongxiu, Liu Di, Shao Jianbo, Peng Xuehua, Yang Yonghong, Liu Zhisheng, Xiang Yun, Zhang Furong, Silva Rona M, Pinkerton Kent E, Shen Kunling, Xiao Han, Xu Shunqing, Wong Gary W K, Chinese Pediatric Novel Coronavirus Study Team (2020). SARS-CoV-2 Infection in Children. N Engl J Med.

[16] WHO Coronavirus (COVID-19) Dashboard.

[17] Yu Y, Li Y, Zhang Z, Gu Z, Zhong H, Zha Q, Yang L, Zhu C, Chen E (2020). A bibliometric analysis using VOSviewer of publications on COVID-19. Ann Transl Med.

[18] Şenel Engin, Topal F (2020). Holistic Analysis of Coronavirus Literature: A Scientometric Study of the Global Publications Relevant to SARS-CoV-2 (COVID-19), MERS-CoV (MERS) and SARS-CoV (SARS). Disaster Med Public Health Prep.

[19] Odone A, Salvati Stefano, Bellini Lorenzo, Bucci Daria, Capraro Michele, Gaetti Giovanni, Amerio Andrea, Signorelli Carlo (2020). The runaway science: a bibliometric analysis of the COVID-19 scientific literature. Acta Biomed.

[20] Patil S (2020). Indian Publications on SARS-CoV-2: A bibliometric study of WHO COVID-19 database. Diabetes Metab Syndr.

[21] Liu N, Chee M, Niu C, Pek P, Siddiqui F, Ansah J, Matchar D, Lam S, Abdullah H, Chan A, Malhotra R, Graves N, Koh M, Yoon S, Ho A, Ting D, Low J, Ong M (2020). Coronavirus disease 2019 (COVID-19): an evidence map of medical literature. BMC Med Res Methodol.

[22] Nowakowska J, Sobocińska Joanna, Lewicki M, Lemańska Żaneta, Rzymski P (2020). When science goes viral: The research response during three months of the COVID-19 outbreak. Biomed Pharmacother.

[23] De Felice Francesca, Polimeni A (2020). Coronavirus Disease (COVID-19): A Machine Learning Bibliometric Analysis. In Vivo.

[24] Mao X, Guo L, Fu P, Xiang C (2020). The status and trends of coronavirus research: A global bibliometric and visualized analysis. Medicine (Baltimore).

[25] Chahrour M, Assi S, Bejjani M, Nasrallah A, Salhab H, Fares M, Khachfe H (2020). A Bibliometric Analysis of COVID-19 Research Activity: A Call for Increased Output. Cureus.

[26] Lou J, Tian S, Niu S, Kang X, Lian H, Zhang L, Zhang J (2020). Coronavirus disease 2019: a bibliometric analysis and review. Eur Rev Med Pharmacol Sci.

[27] Warin T (2020). Global Research on Coronaviruses: An R Package. J Med Internet Res.

[28] Fidahic M, Nujic D, Runjic R, Civljak M, Markotic F, Lovric Makaric Zvjezdana, Puljak L (2020). Research methodology and characteristics of journal articles with original data, preprint articles and registered clinical trial protocols about COVID-19. BMC Med Res Methodol.

[29] Serlachius A, Badawy SM, Thabrew H (2020). Psychosocial Challenges and Opportunities for Youth With Chronic Health Conditions During the COVID-19 Pandemic. JMIR Pediatr Parent.

[30] Badawy SM, Radovic A (2020). Digital Approaches to Remote Pediatric Health Care Delivery During the COVID-19 Pandemic: Existing Evidence and a Call for Further Research. JMIR Pediatr Parent.

[31] Brown A, Horton R (2020). A planetary health perspective on COVID-19: a call for papers. The Lancet.

[32] Ioannidis J (2020). Coronavirus disease 2019: The harms of exaggerated information and non-evidence-based measures. Eur J Clin Invest.

