# Supplementary figures and images for: A Global Overview of COVID-19 Research in the Pediatric Field: Bibliometric Review
Source: JMIR Pediatr Parent. 2021 Jul 23;4(3):e24791. doi: 10.2196/24791 (PMC8315163; doi:10.2196/24791)

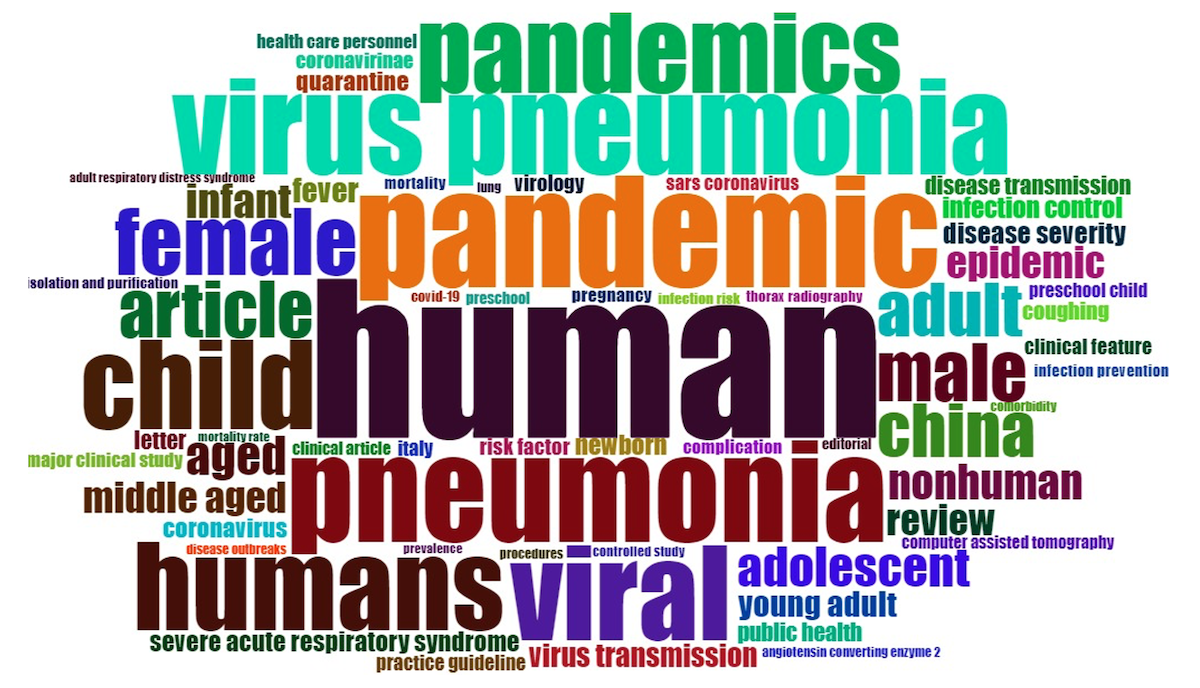

Supplement: Multimedia Appendix 1 [file pediatrics_v4i3e24791_app1.png]
